# Effect of Vision Therapy on Accommodation in Myopic Chinese Children

**DOI:** 10.1155/2016/1202469

**Published:** 2016-12-21

**Authors:** Martin Ming-Leung Ma, Mitchell Scheiman, Cuiyun Su, Xiang Chen

**Affiliations:** ^1^State Key Laboratory of Ophthalmology, Zhongshan Ophthalmic Center, Sun Yat-sen University, Guangzhou 510060, China; ^2^Pennsylvania College of Optometry, Salus University, Philadelphia, PA, USA

## Abstract

*Introduction*. We evaluated the effectiveness of office-based accommodative/vergence therapy (OBAVT) with home reinforcement to improve accommodative function in myopic children with poor accommodative response.* Methods*. This was a prospective unmasked pilot study. 14 Chinese myopic children aged 8 to 12 years with at least 1 D of lag of accommodation were enrolled. All subjects received 12 weeks of 60-minute office-based accommodative/vergence therapy (OBAVT) with home reinforcement. Primary outcome measure was the change in monocular lag of accommodation from baseline visit to 12-week visit measured by Shinnipon open-field autorefractor. Secondary outcome measures were the changes in accommodative amplitude and monocular accommodative facility.* Results*. All participants completed the study. The lag of accommodation at baseline visit was 1.29 ± 0.21 D and it was reduced to 0.84 ± 0.19 D at 12-week visit. This difference (−0.46 ± 0.22 D; 95% confidence interval: −0.33 to −0.58 D) is statistically significant (*p* < 0.0001). OBAVT also increased the amplitude and facility by 3.66 ± 3.36 D (*p* = 0.0013; 95% confidence interval: 1.72 to 5.60 D) and 10.9 ± 4.8 cpm (*p* < 0.0001; 95% confidence interval: 8.1 to 13.6 cpm), respectively. * Conclusion*. Standardized 12 weeks of OBAVT with home reinforcement is able to significantly reduce monocular lag of accommodation and increase monocular accommodative amplitude and facility. A randomized clinical trial designed to investigate the effect of vision therapy on myopia progression is warranted.

## 1. Introduction

Myopia has become a significant public health problem around the world [1]. Researchers have suggested that various oculomotor factors may be related to the development, progression, and stabilization of myopia including poor accommodative response [2–8], decreased accommodative tonus [9], decreased accommodative amplitude [10], reduced accommodative facility [11–13], increased accommodative adaptation [14], increased accommodative variability [15], near phoria [16], and AC/A ratio [8, 17, 18]. Both animal [19, 20] and human [3, 21] research have led to the development of a theory referred to as the “blur hypothesis.” This theory suggests that retinal defocus caused by underaccommodation may be a factor related to myopia development and progression [3, 21].

Based on this theory, bifocals and progressive addition lenses [22–24] (PALs) have been proposed as treatments to slow the progression of myopia. Studies to investigate these treatments have shown statistically significant but not clinically meaningful effect in the general myopia population. However, larger effects have been found in children with poor accommodative response and esophoria at near [23, 24], but still only borderline clinically meaningful. A potential reason for the minimal success of bifocals and PALs is that neither approach is designed to eliminate the underlying accommodative disorder. Rather, both treatments are compensatory and designed to allow the child to achieve clear retinal focus in spite of poor accommodative response. However, simply prescribing a bifocal or PAL does not guarantee that there will be a clear retinal image. If a child does not wear the glasses or does not use the reading segment or if the glasses are not adjusted or worn properly, the child may continue to experience a significant amount of retinal defocus. These problems may be factors associated with the disappointing treatment effects in recent studies.

Vision therapy has been proposed [25–31] as a treatment option to improve accommodative function in adults and children with accommodative disorders. If such treatment is successful leading to a more accurate accommodative response, there would be less retinal defocus than expected with PALs, potentially leading to a larger treatment effect for myopia progression.

The available literature on improving accommodative function with accommodative or vergence therapy includes a number of studies [25–31], all of which have limitations in methodology including small sample size, retrospective study design, unmasked examiners, and other potential design features that could introduce bias. Recently, there is a well-designed randomized clinical trial with placebo therapy group [25] investigating effectiveness of vision therapy for accommodation in children with accommodative dysfunction which were defined as having decreased accommodative amplitude with respect to age, accommodative facility, or both and it showed significant improvement in amplitude and facility. Unfortunately the lag of accommodation was not measured in this study. In addition there is another recent randomized clinical trial [26] that studied the relationship between vision therapy and lag of accommodation in young adults. Although their data suggest vision therapy cannot change the lag of accommodation, there were some limitations in their study design that may have affected the outcome. Thus, further study of the potential for vision therapy to improve accommodative function and potentially slow the progression of myopia is warranted. However, the first step is to determine whether vision therapy can improve accommodative function, particularly accommodative lag.

The objective of this pilot study is to evaluate the effectiveness of office-based accommodative/vergence therapy with home reinforcement to improve accommodative function in myopic children with poor accommodative response.

## 2. Methods

### 2.1. Participants

Data were collected at the Zhongshan Ophthalmic Center at Guangzhou, China. All procedures met the tenets of the Declaration of Helsinki and were approved by the medical ethics committee of Zhongshan Ophthalmic Center. Written consent and assent were obtained from participants. This study was registered at ClinicalTrials.gov (identifier: NCT02578407).

Children were recruited and enrolled from September 2015 to November 2015 at the Clinical Research Center of Zhongshan Ophthalmic Center. Potential participants with eligible age were identified from the database; then they were invited for the study. The inclusion and exclusion criteria are listed as follows.

*Inclusion Criteria*

- 8 to 12 years old
−0.75 D to −4.50 D spherical equivalent by cycloplegic autorefraction in both eyes
- Astigmatism ≤ 1.50 D in both eyes
- Anisometropia ≤ 1.00 D
- Lag of accommodation ≥ 1.00 D spherical equivalent at 33 cm in right eye by noncycloplegic autorefraction
- Visual acuity correctable to 0.8 or better in each eye.

*Exclusion Criteria*

- Current or prior use of PALs, bifocals, or contact lenses in either eye (prior or current use of SVLs allowed)
- Previous history of vision therapy
- History of strabismus, amblyopia, or nystagmus
- History of diabetes or seizures
- History of any ocular, systemic, or neurodevelopmental condition that might influence refractive development
- Use of ocular or systemic medications containing atropine, pirenzepine, or antiepileptic medications in recent 3 months
- History of any ocular surgery that might influence refractive development
- Developmental disability, attention deficit hyperactivity disorder (ADHD), or learning disability diagnosis in children that in the investigator's discretion would interfere with office-based treatment
- Relocation anticipated for 1 year
- Birth weight lower than 1250 grams (2 lbs, 12 oz)
- Siblings in the study
- History of hyperphoria

### 2.2. Screening Visit and Baseline Visit

At the screening visit, traditional optometric tests including lensometry, autorefraction, visual acuity, and the cover test were performed.

If participants' habitual monocular distance VA was 6/7.5 or better in both eyes at the screening visit, written consent and assent were obtained on the same day and baseline vision examination was performed. The baseline testing included Convergence Insufficiency Symptom Survey (CISS) questionnaire [32], visual acuity, cover test at distance and near, accommodative amplitude and facility, Monocular Estimation Method (MEM) retinoscopy [33], objective assessment of lag of accommodation, near point of convergence, step vergence, vergence facility, and a cycloplegic refraction using 1% Tropicamide.

If participants' habitual monocular distance VA was poorer than 6/7.5 in either eye at the screening visit, cycloplegic refraction using 1% Tropicamide was performed on the same day. Glasses were prescribed and the participants were reexamined after wearing the new prescription for 2 weeks. At the reevaluation, lag of accommodation and lensometry were performed again to make sure all inclusion criteria were satisfied. Then written consent and assent were obtained on the same day and the baseline vision examination was performed except for the cycloplegic refraction.

All newly prescribed glasses or habitual glasses used by the participants had to fulfill the following criteria: (1) Spherical equivalent anisometropia must be <0.75 D of the full anisometropic correction; (2) astigmatism must be <0.75 D of full correction; axis must be within 6° if astigmatism ≥ 1.00 D; (3) for myopia, the spherical equivalent must be <0.75 D of the full myopic correction.

Participants needed to wear the prescribed glasses for all visits to ensure that the vergence and accommodative demand during therapy and testing remained the same.

### 2.3. Clinical Tests Procedures

The lag of accommodation was measured in the right eye only using a Shin-Nippon open-field autorefractor (NVISION-K 5001) with participant wearing their up-to-date glasses. A vertical row of printed 20/30 optotypes (Gulden fixation target) was placed 33 cm from participant (3 D accommodative demand). During the measurement, the investigator frequently reminded the participant to keep the target clear. An average of 10 successive measurements of lag was obtained. Measurements were taken with a precision level of 0.25 D. Invalid readings (difference in spherical equivalent/sphere/cylinder > 0.75 D within a set of data) were excluded and the test was redone until valid readings were obtained. The vertex distance for the eyeglass was assumed to be 12 mm for all measurements. Lens effectivity and residual refractive error were adjusted using the formula described in a previous study [34].

All of the following tests were performed using a 20/30 column of letters as the fixation target. The monocular accommodative amplitude (OD only) was repeated three times using the Astron Accommodative Rule. The patient was instructed to report the “first sustained blur” as the target was moved slowly (2 cm/sec) towards the eye. Accommodative facility was measured (OD only) with a ±2.00 diopters flipper. Participants were instructed to report clarity (say “clear”) as soon as the letters were clear. The number of flips per minute was recorded. Fusional vergence was measured using a horizontal prism bar and vergence facility was assessed with a 12BO/3BI flipper. The cover test was used to determine the distance and near phoria, and the near point of convergence (NPC) was measured with the Astron Accommodative Rule. Cycloplegic objective refraction was measured by Topcon KR-8900 autorefractor.

### 2.4. Treatment

All subjects received 12 weeks of office-based accommodative/vergence therapy (OBAVT) with home reinforcement administered by a trained therapist during a 60-minute weekly office visit, combined with procedures to practice at home, for 15 minutes, five times per week. The treatment sequence proposed for this study was very similar to a treatment protocol previously used in the Convergence Insufficiency Treatment Trial (CITT), randomized clinical trial [35]. We modified this protocol to focus more on accommodative procedures and less on convergence therapy as suggested in vision therapy textbooks [36].

### 2.5. Follow-Up Visit

The follow-up visits were at the 6-week visit and 12-week visit. All of the baseline accommodative and vergence measurements were repeated at these visits.

### 2.6. Primary and Secondary Outcome Measures

The primary outcome measure in this study was the change in lag of accommodation from baseline visit to 12-week visit. Secondary outcome measures were the change in accommodative amplitude and monocular accommodative facility.

### 2.7. Statistical Analysis

Two-tailed paired *t*-test (90% power and 5% significance level) was used to test the significance of change in outcome measures. Confidence intervals and Cohen's *d* score effect size were calculated for primary and secondary outcome measures.

## 3. Results

27 Chinese children were screened and 14 of them enrolled into this pilot study. Potential participants were excluded primarily due to refractive error related reasons. All participants completed the study. All participants attended 12 weeks of vision therapy and the average time required from baseline visit to 12-week visit was 13.8 ± 2.6 weeks. No adverse events occurred. The baseline characteristics were shown in Table 1.

### 3.1. Monocular Lag of Accommodation

**Figure 1:**
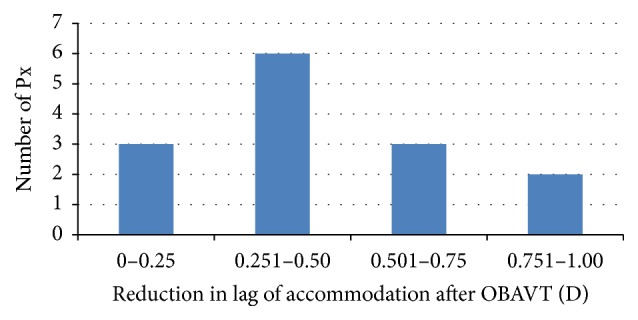
Distribution of reduction in lag of accommodation after vision therapy.

The results for the monocular lag of accommodation are illustrated in Table 2. All participants showed a decrease in lag of accommodation with a mean reduction of 0.46 ± 0.22 D (*p* < 0.0001; 95% confidence interval: 0.33 to 0.58 D; Cohen's *d* effect size: 2.25) from baseline visit to 12-week visit. There were statistically significant differences between the lag at the 6-week visit and the baseline visit (*p* = 0.0026) and between the 12-week visit and the baseline visit (*p* < 0.001). Although the mean lag continued to decrease from the 6-week visit to the 12-week visit, this change was not statistically significant (*p* = 0.13). Two-thirds of the improvement in lag occurred in the first 6 weeks of therapies (0.31 D decrease). The distribution of reduction in lag of accommodation after vision therapy is demonstrated in Figure 1.

**Figure 2:**
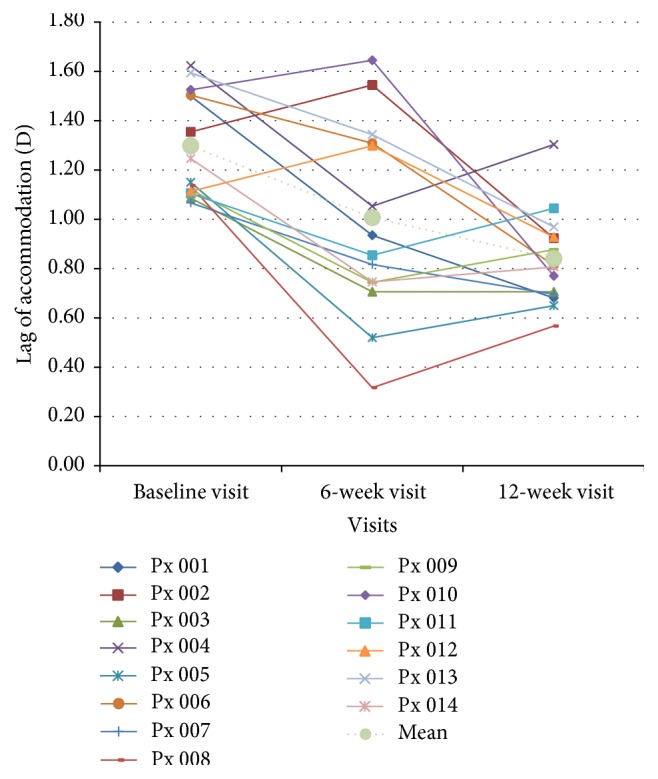
Lag of accommodation over time. The dotted line is the mean data while others are data of individual participant.

The kinetics of the improvement in lag can be divided into 3 groups and are illustrated in Figure 2. In the first group (participants 002, 010, and 012), the lag increased at 6-week visit but decreased at the 12-week visit. However, by week 12 all three had a lower lag than at baseline visit. In the second group (participants 004, 005, 008, 009, and 014), the lag was reduced at 6-week visit and then increased after the last 6 weeks of vision therapy. In the third group (participants 001, 003, 006, 007, 011, and 013), the lag was reduced at 6-week visit and then either stabilized or further decreased at 12-week visit.

### 3.2. Monocular Accommodative Amplitude

The results for the monocular accommodative amplitude are illustrated in Table 3. Although all participants started the study with a normal accommodative amplitude (defined as >2.00 D below the lowest expected amplitude based on Hofstetter's formula of 15-1/4 age [37, 38]), the amplitude increased from 16.86 ± 3.01 D at baseline visit to 20.52 ± 3.20 D at 12-week visit (*p* = 0.0013; 95% confidence interval: 1.72 to 5.60 D; Cohen's *d* effect size: 1.17). There were significant differences between the 6-week visit and the baseline visit (*p* < 0.0001) and between the 12-week visit and the baseline visit but not between 6-week visit and the 12-week visit (*p* = 0.86). The improvement in amplitude occurred during the first 6 weeks (3.77 D increase) but not during the last 6 weeks of therapy (0.11 D decrease).

### 3.3. Monocular Accommodative Facility

The results for the monocular accommodative facility are illustrated in Table 4. Seven participants had a reduced facility at baseline (defined as <6 cpm which is 1 standard deviation below the normative value for school age children [39–41]). Facility increased from 6.9 ± 4.1 cpm at baseline visit to 17.8 ± 6.1 cpm at 12-week visit (*p* < 0.0001; 95% confidence interval: 8.1 to 13.6 cpm; Cohen's *d* effect size: 2.10). In contrast to lag and amplitude, the differences between the baseline visit and 6-week visit (*p* = 0.00023), 6-week visit and the 12-week visit (*p* = 0.00027), and the baseline visit and the 12-week visit were all statistically significant. The improvement in facility is of similar level in both the first 6 weeks of therapies (5.7 cpm increase) and the last 6 weeks of therapies (5.1 cpm increase).

### 3.4. Change in Other Clinical Measures

There were significant changes in a number of clinical measures except for the MEM and refraction from baseline to the 12-week visit as illustrated in Table 5.

### 3.5. Post Hoc Sample Size Calculation

Based on the difference and standard deviation of the primary outcome measure which is the lag of accommodation, 5 participants are enough for detecting a significant change by two-tailed paired *t*-test with 90% power and 5% significance level.

## 4. Discussion

In this pilot study, we found statistically significant and clinically meaningful improvement in monocular lag of accommodation, monocular accommodative amplitude, and facility after 12 weeks of OBAVT in Chinese myopic children with poor accommodative response. These data suggest OBAVT with home reinforcement is an effective method of improving the accommodation in this population.

### 4.1. Lag of Accommodation

In a recent report [26] on the effect of vision therapy on lag of accommodation, the authors did not find a significant change in lag. There are a number of differences in study design between the current study and the previous one. In their study, the therapy was home-based and only included one lens flipper exercise at near. Participants were asked to perform the same technique for 18 minutes per day for up to 6 weeks. It is possible that their subjects lost interest because of this lack of variability in vision therapy procedures. This may have affected compliance and also only the facility aspect of the accommodative system was trained. In contrast, in this study participants attended office-based therapy monitored by trained/experienced clinicians and included a wide variety of procedures. There is also another study with a smaller sample size [28] that reported that vision therapy did not improve the lag but it suffers from the same limitation of only using lens flipper exercise at near.

Cohen's *d* effect size of the difference in lag before and after OBAVT was 2.25. Using Cohen's [42, 43] guidelines for interpretation of effect size (0.2 is small, 0.5 is medium, and 0.8 is large), this is considered to be a large improvement and is clinically meaningful. We are unaware of a previous study which has a primary objective of finding the normative data of lag of accommodation measured by autorefraction in general pediatric population. As the norm of Nott dynamic retinoscopy at the average working distance of children (25 cm) [44] is +0.30 ± 0.39 D [45], a priori we suggested that a 0.25 D change in lag measurement by autorefraction should be regarded as clinically meaningful. Eleven participants (79%) achieved such a change.

While there was a statistically and clinically significant decrease in the objective measure of lag, the MEM finding showed an insignificant increase. In a previous study [46], the MEM has been shown to be in poor agreement with objective open-field autorefractor although its agreement with other methods like near red-green duochrome and dynamic cross-cylinder is good. A previous study [47] demonstrated a good agreement between the autorefractor and Nott technique which does not require any supplementary lenses for evaluation of the lag. It is possible that the MEM data is contaminated when the additional lens is inserted into patient's line of sight [46]. We believe objective autorefractor assessment may provide more accurate and consistent data than MEM testing.

Regarding the kinetics of the change in lag, in a large proportion of the participants (11 out of 14) the lag improved after only 6 weeks of therapy. Moreover, considering the statistically insignificant difference between the lag at 6-week visit and 12-week visit, one might conclude that 6 weeks of therapy may be enough but we suggest caution because of the small sample size. In addition, considering that a vision therapy program for accommodative insufficiency can take up to 24 office visits [36], further improvement in lag may occur with more visits.

### 4.2. Amplitude and Facility

In addition to the improvement of accommodative error, this study showed that vision therapy can improve the amplitude [25, 28, 29] and facility [25, 26, 29] consistent with previous reports. Amplitude [10] and facility [11–13] have been proposed to be related to myopia progression individually, so an improvement in these areas might be beneficial if vision therapy is used to slow the progression of myopia.

Regarding accommodative amplitude, our data demonstrated that even participants with clinically normal amplitude can gain a significant improvement after vision therapy. However, the possibility of placebo effect or effect from participants' desire to please the examiner should be carefully considered.

### 4.3. Strength and Weakness of the Study

The strengths of our study include its prospective design, the use of objective measures of lag of accommodation, and participants wearing the refractive correction from baseline to the last visit which minimize the effect of spectacle adaptation.

Although the sample size of this pilot study was small, post hoc testing suggests that it was sufficient considering the large effect size found with the primary outcome measure. Other limitations of study include lack of control group and masking of both examiners and participants.

### 4.4. Potential Clinical Implication

The result of this pilot study suggests that accommodative function can be improved in myopic children with poor accommodative response. Among the three aspects of accommodative system we evaluated, lag of accommodation is the most widely studied due to its hypothesized relationship to retinal image defocus and myopia progression [2–6, 8]. If lag of accommodation is a factor related to myopia progression, one could hypothesize that OBAVT may slow the rate of myopia progression, provided that this improvement in lag is sustainable and transferrable to daily near task. A previous study [27] investigating treatments to slow the progression of myopia included vision therapy as a treatment arm did not find that vision therapy was an effective method to slow myopia progression. However, that study used only home-based therapy and did not report on compliance with the home-based therapy. In addition, the age range of their subjects was 14–22 with a mean age of about 16 years. In the future, it would be desirable to investigate the effect of vision therapy on myopia progression in a younger cohort of myopic children whose myopia is more likely to be actively progressing.

## 5. Conclusion

In conclusion, this pilot study showed that standardized 12 weeks of office-based accommodative/vergence therapy with home reinforcement is able to significantly reduce monocular lag of accommodation and increase monocular accommodative amplitude and facility. A randomized clinical trial designed to investigate the effect of vision therapy on myopia progression is warranted.

## Figures and Tables

**Table 1:** Baseline characteristics.

Characteristic	Value
Male sex, *n* (%)	7 (50%)
Age, mean ± SD	9.4 ± 1.4
Cycloplegic objective refraction, D	
Right eye, mean ± SD	−2.61 ± 1.00
Left eye, mean ± SD	−2.75 ± 0.95
Exophoria, Δ	
Distance, mean ± SD	0.6 ± 1.7
Near, mean ± SD	2.3 ± 4.8

**Table 2:** Result and comparison of lag of accommodation values at different visits.

Px	Lag of accommodation (D)			Change from baseline visit to 6-week visit		Change from 6-week visit to 12-week visit		Change from baseline visit to 12-week visit	
	Baseline visit	6-week visit	12-week visit	In diopter	in%	In diopter	in%	In diopter	in%
001	1.50	0.94	0.68	−0.56	−37.7%	−0.25	−27.2%	−0.82	−54.6%
002	1.35	1.54	0.92	0.19	14.0%	−0.62	−40.2%	−0.43	−31.8%
003	1.09	0.71	0.71	−0.38	−35.0%	0.00	0.0%	−0.38	−35.0%
004	1.62	1.05	1.30	−0.57	−35.1%	0.25	23.7%	−0.32	−19.7%
005	1.15	0.52	0.65	−0.63	−54.8%	0.13	24.9%	−0.50	−43.5%
006	1.50	1.31	0.81	−0.20	−13.0%	−0.50	−37.9%	−0.69	−45.9%
007	1.07	0.82	0.70	−0.25	−23.4%	−0.12	−14.7%	−0.37	−34.7%
008	1.14	0.32	0.57	−0.82	−72.1%	0.25	78.9%	−0.57	−50.1%
009	1.12	0.74	0.88	−0.37	−33.5%	0.13	17.8%	−0.24	−21.7%
010	1.52	1.64	0.77	0.12	7.9%	−0.87	−53.2%	−0.75	−49.5%
011	1.10	0.85	1.04	−0.25	−22.6%	0.19	22.2%	−0.06	−5.4%
012	1.11	1.30	0.93	0.19	16.6%	−0.37	−28.5%	−0.18	−16.6%
013	1.59	1.34	0.97	−0.25	−15.7%	−0.37	−27.9%	−0.62	−39.2%
014	1.25	0.75	0.81	−0.50	−40.1%	0.06	8.1%	−0.44	−35.3%

Mean	1.29	0.99	0.84	−0.31	−23.7%	−0.15	−15.2%	−0.46	−35.3%
SD	0.21	0.39	0.19	0.31		0.35		0.22	

**Table 3:** Result and comparison of monocular accommodative amplitudes at different visits.

Px	Accommodative amplitude (D)			Change from baseline visit to 6-week visit (D)	Change from 6-week visit to 12-week visit (D)	Change from baseline visit to 12-week visit (D)
	Baseline visit	6-week visit	12-week visit			
001	15.00	21.43	20.00	6.43	−1.43	5.00
002	19.35	18.75	19.35	−0.60	0.60	0.00
003	18.18	21.43	20.00	3.25	−1.43	1.82
004	23.08	27.27	26.09	4.20	−1.19	3.01
005	16.67	25.00	25.00	8.33	0.00	8.33
006	16.67	21.43	24.00	4.76	2.57	7.33
007	16.67	23.08	18.18	6.41	−4.90	1.52
008	15.38	18.75	16.67	3.37	−2.08	1.28
009	12.50	21.43	21.43	8.93	0.00	8.93
010	15.79	20.00	22.22	4.21	2.22	6.43
011	13.33	16.67	19.35	3.33	2.69	6.02
012	16.22	16.22	14.63	0.00	−1.58	−1.58
013	22.22	20.69	22.22	−1.53	1.53	0.00
014	15.00	16.67	18.18	1.67	1.52	3.18

Mean	16.86	20.63	20.52	3.77	−0.10	3.66
SD	3.01	3.16	3.20	3.15	2.15	3.36

**Table 4:** Result and comparison of monocular accommodative facilities at different visits.

Px	Accommodative facility (cpm)			Change from baseline visit to 6-week visit (cpm)	Change from 6-week visit to 12-week visit (cpm)	Change from baseline visit to 12-week visit (cpm)
	Baseline visit	6-week visit	12-week visit			
001	5	10	17	5	7	12
002	11	14	20	3	6	9
003	5	16	26	11	10	21
004	10	10	16	0	6	6
005	8	20	21	12	1	13
006	9	17	15	8	−2	6
007	9	14	25	5	11	16
008	5	6	13	1	7	8
009	2	16	17	14	1	15
010	9	14	25	5	11	16
011	4	11	14	7	3	10
012	0	1	5	1	4	5
013	4	8	11	4	3	7
014	16	20	24	4	4	8

Mean	6.9	12.6	17.8	5.7	5.1	10.9
SD	4.1	5.4	6.1	4.3	3.9	4.8

**Table 5:** Effect of vision therapy on other parameters.

	Baseline visit	12-week visit	Difference	*p* value
CISS score	10.6	6.7	−3.9	0.02382
SD	5.5	5.1	5.6	

NPC break point (cm)	3.3	2.2	−1.1	0.00256
SD	1.2	0.5	1.1	

NPC recovery point (cm)	5.4	3.2	−2.1	0.00017
SD	1.4	0.8	1.5	

Vergence facility (cpm)	9.4	15.5	6.1	0.00111
SD	3.2	6.3	5.5	

Near step NFV break point (Δ)	17.7	23.5	5.8	0.03603
SD	7.2	6.9	9.3	

Near step NFV recovery point (Δ)	13.0	18.1	5.2	0.03828
SD	7.1	5.7	8.4	

Near step PFV break point (Δ)	39.3	49.6	10.4	0.00441
SD	11.1	0.7	11.3	

Near step PFV recovery point (Δ)	28.7	44.5	15.8	0.00070
SD	13.0	1.0	13.4	

MEM (D)	0.55	0.63	0.07	0.33556
SD	0.20	0.36	0.27	

Cycloplegic objective Rx RE (D)	−2.61	−2.72	−0.11	0.09867
SD	1.00	1.01	0.23	

Cycloplegic objective Rx LE (D)	−2.75	−2.84	−0.09	0.25599
SD	0.95	6.27	0.28	
